# Transformational Leadership and Its Impact on Job Satisfaction and Personal Mastery for Nursing Leaders in Healthcare Organizations

**DOI:** 10.3390/nursrep14040260

**Published:** 2024-11-18

**Authors:** Ippolito Notarnicola, Blerina Duka, Marzia Lommi, Eriola Grosha, Maddalena De Maria, Laura Iacorossi, Chiara Mastroianni, Dhurata Ivziku, Gennaro Rocco, Alessandro Stievano

**Affiliations:** 1 Centre of Excellence for Nursing Scholarship, OPI, 00146 Rome, Italy; gennaro.rocco@uniroma2.it (G.R.); alessandro.stievano@unime.it (A.S.); 2 Faculty of Medicine, University “Our Lady of the Good Counsel”, 1000 Tirana, Albania; bleriduka@yahoo.it; 3 Department of Clinical and Molecular Medicine, Faculty of Medicine and Psychology, University of Rome “La Sapienza”, 00157 Rome, Italy; marzia.lommi@uniroma1.it; 4 University of Rome “Tor Vergata”, 00133 Rome, Italy; e.grosha8400@stud.unizkm.al; 5 Link Campus University, 00165 Rome, Italy; m.demaria@unilink.it (M.D.M.); l.iacorossi@unilink.it (L.I.); c.mastroianni@unilink.it (C.M.); 6 Department of Health Professions, Fondazione Policlinico Universitario Campus Bio-Medico, 00128 Rome, Italy; d.ivziku@policlinicocampus.it; 7 Department of Clinical and Experimental Medicine, University of Messina, 98122 Messina, Italy

**Keywords:** transformational leadership, healthcare organizations, job satisfaction, personal mastery, leadership styles, employee engagement

## Abstract

Background: Transformational leadership fosters trusting relationships; new visions; and personal, professional, and cultural growth. Effective leaders support their team’s motivational growth and organizational goals. This study aims to underscore the importance of transformational leadership and its various dimensions, focusing on its impact on job satisfaction and personal mastery among nursing leaders in healthcare organizations. Method: A cross-sectional design with convenience sampling was used. The evaluation tools included the Multifactor Leadership Questionnaire (MLQ-6S), the Satisfaction of Employees in Health Care (SEHC) questionnaire, and the Personal Mastery Scale (PMS). Results: The findings indicate that job satisfaction is influenced by transformational leadership, emphasizing the importance of tailored leadership development strategies within healthcare organizations. The laissez-faire leadership style was the only one showing no correlation with nurses’ job satisfaction. Other leadership styles showed significant positive or negative correlations with the analyzed variables. Conclusions: Transformational leaders are essential for fostering trust and enhancing job satisfaction in healthcare settings. Positive leadership styles contribute to higher levels of job satisfaction and personal mastery among nursing leaders. Conversely, laissez-faire and autocratic leadership styles can negatively impact performance and staff satisfaction. These findings highlight the critical role of leaders in creating positive work environments and supporting employee development and well-being in healthcare.

## 1. Introduction

In the ever-evolving landscape of the healthcare sector, transformational leadership emerges as a crucial area of interest for scholars and practitioners alike. This leadership approach focuses not only on the skills and commitment of organizational members but also on the strategies and processes through which they can be valued and developed through the influence of leaders [1,2].

Transformational leadership is distinguished by its ability to change and transform individuals, urging them to surpass their limits and achieve outcomes beyond expectations [3,4]. At the core of this process lies an emphasis on values, emotions, morality, and long-term goals, with particular attention to satisfying collaborators’ needs and recognizing them as unique individuals [5,6,7].

Transformational leadership is realized through negotiation and the exchange of ideas and goals between leaders and collaborators. It aims to achieve organizational objectives and foster a culture of growth and development [8]. The motivation of organizational members is fueled by the recognition of success, tangible rewards, or punishment in case of failure [9].

Moreover, in nursing, transformational leadership plays a vital role in shaping job satisfaction and fostering personal mastery among nursing professionals. A comprehensive job description aligned with transformational leadership principles can enhance job satisfaction by providing clarity, autonomy, and opportunities for professional growth [10]. Personal mastery, defined in this study as the continuous improvement of skills and competencies in both personal and professional contexts, is fostered by transformational leaders who encourage learning, innovation, and self-development among their team members. While the concept of personal mastery used in this research broadly encompasses individual growth, it may not fully capture specific workplace dynamics, which is acknowledged as a limitation [11,12].

However, the current challenge lies in providing increasingly personalized, reliable, and technologically advanced patient care, ensuring a prompt treatment response and efficient coordination to enhance the quality of nursing care [13,14]. Continuous innovation in hospitals is essential for improving the patient experience and promoting the development of the nursing community [15]. Effective leadership is a fundamental catalyst for fostering innovation among nurses, yet our understanding of the mechanisms that stimulate innovative work behaviors remains limited [16].

Despite the widespread use of transformational leadership in nursing care, evidence of its effectiveness in nursing innovation is still being clearly determined, and previous study conclusions are often contradictory [17,18]. This lack of coherence is influenced by mediating factors, which play a crucial role in the relationship between transformational leadership and nursing innovation behavior but have been largely overlooked in research [19].

This study aims to underscore the importance of transformational leadership and its various dimensions, focusing on its impact on job satisfaction and personal mastery among nursing leaders in healthcare organizations. The research site was chosen because it is representative of leadership dynamics in the Italian healthcare sector, with a specific focus on transformational leadership and its impact on nursing team performance. In particular, the hospital (or organization) in which this study was conducted is an exemplary model of the implementation of innovative leadership approaches. The site was also selected to explore how cultural and organizational factors influence the effectiveness of leadership in the healthcare context.

This study’s objectives are as follows: to investigate the link between transformational leadership, job satisfaction, and personal mastery among nursing leaders, focusing on how transformational leadership influences personal and professional growth within healthcare organizations.

The aspect of this study focuses on the relationship between transformational leadership, job satisfaction, and personal mastery among nursing leaders. Specifically, it examines how transformational leadership can positively influence staff motivation and enhance the quality of care provided to patients. This aspect is central to this study as we believe that effective leadership is crucial for the professional growth and satisfaction of nursing leaders within healthcare organizations.

## 2. Materials and Methods

This study employed a cross-sectional design with a convenience sampling method. The researchers ensured robustness and quality throughout this study by adhering to the Strengthening Reporting of Observational Studies in Epidemiology (STROBE) guidelines [20]. Data collection spanned from 30 June 2023 to 30 November 2023. Participants were included if they were nursing leaders with at least three years of experience in a healthcare setting. The participants were recruited through email invitations and internal announcements within the selected healthcare organizations, targeting nursing leaders with the necessary qualifications and experience for their study. The exclusion criteria included those in administrative roles without direct leadership responsibilities. The participants were recruited through a few recruitment methods, e.g., email invitations, or internal announcements within the selected healthcare organizations. A power analysis was conducted to determine the necessary sample size for reliable statistical analysis. Based on an expected effect size of 0.30 (medium effect), a significance level of 0.05, and a power of 0.80, the required sample size was calculated to ensure sufficient statistical power. The power analysis indicated a minimum sample size of 175 subjects to achieve adequate statistical power, considering 25 subjects for each of the seven independent variables. However, the sample of 37 participants was deemed acceptable for the exploratory purposes of this study, with an acknowledgment of the reliability limitations associated with the smaller sample size.

The data were analyzed using SPSS, version 26 (IBM Corporation, Armonk, NY, USA). Descriptive statistics, correlation analysis, and regression analysis were performed to examine the relationships between transformational leadership, job satisfaction, and personal mastery. These instruments were meticulously chosen to gauge the prevalent leadership styles among a cohort of middle and senior nursing leaders within a hospital situated in the Lazio region.

### 2.1. Population

The study population consisted of nurses and healthcare professionals employed in public and private hospitals across Italy. The participants were selected based on their professional roles, with a focus on individuals who had at least three years of experience in direct patient care. This selection ensured a diverse sample that reflects the target population in terms of gender, years of experience, and regional representation. The population was chosen to provide insights into the impact of transformational leadership on job satisfaction and personal mastery in healthcare settings.

### 2.2. Data Analysis

The data were analyzed using descriptive statistics, correlation analysis, and regression analysis to examine relationships between transformational leadership, job satisfaction, and personal mastery. We employed SPSS software to ensure accurate and consistent results. The analysis focused on identifying significant relationships between transformational leadership practices and the outcomes of interest. The data were interpreted according to this study’s objectives, and we ensured the robustness of the analysis through cross-validation methods.

### 2.3. Ethical Statement

Before data collection, the participants were informed about this study’s purpose, procedures, research method, data anonymity, and the option to withdraw at any time without consequences. The participants were provided with full information about this study, including its purpose, procedures, potential risks, and benefits. Informed consent was obtained explicitly. The participants were asked to sign a consent form before completing the questionnaires. Only those who provided signed consent were allowed to proceed with this study. This process ensured that all the participants understood their involvement and agreed to participate voluntarily. Ethical approval for this study was obtained from the Ethics Committee of the Center of Excellence for Nursing Culture and Research (CECRI) (Protocol No. 2.21.27).

### 2.4. Multifactor Leadership Questionnaire, Form 6S (MLQ-6S)

The questionnaire used in this study is the Multifactorial Leadership Questionnaire, Form 6S (MLQ-6S), developed by, Avolio and Bass (2004) [21]. The MLQ-6S is a leadership assessment tool based on research across multiple disciplines, and its validity and reliability have been demonstrated.

The MLQ-6S questionnaire consists of twenty-one items to measure three different leadership styles: transformational leadership (TL), transactional leadership (TAL) (contingent reward), and passive/avoidant leadership (LF) (passive management by exception, laissez-faire), in seven dimensions. Each item measures one of three leadership styles, using a 5-point Likert scale (0 = not at all to 4 = frequently, if not always). The scores of the three items, under the seven sub-themes, are then combined to be ranked into high (9–12), moderate (5–8), and low (1–4). In addition, some sociodemographic questions, such as age, gender, and education level, were added to the MLQ-6S.

### 2.5. Satisfaction of Employees in Health Care (SEHC)

The “Satisfaction of Employees in Health Care (SEHC)” tool consists of 20 items and has been used to assess the level of employee satisfaction in the healthcare sector [22]. The questionnaire uses a 5-point Likert scale, ranging from “Strongly Disagree” to “Strongly Agree” for the first 19 items. The response for item 19 is rated on a binary scale of “Probably No” and “Definitely No” for “No” and “Probably Yes” and “Definitely Yes” for “Yes”. Item 20 is rated on a 10-point scale, ranging from “Worst” to “Best”. The questionnaire explores different aspects of work and the work environment, including management support, work–life balance, professional development opportunities, remuneration, relationship with colleagues, and perception of the effectiveness of organizational policies. The participants’ responses were collected and analyzed to assess the overall level of employee satisfaction in the healthcare setting. The SEHC tool has demonstrated good-to-excellent internal consistency, with Cronbach’s alpha coefficients of 0.70 or higher for its three main factors: relationships with management and supervisors, job content, and relationships with coworkers. Additionally, the tool showed strong construct validity through factor analysis and moderate convergent validity in a healthcare setting [22].

### 2.6. The Personal Mastery Scale (PMS)

The Personal Mastery Scale [23] was developed to assess individuals’ perceptions of control and influence over their lives. This scale revolves around “mastery,” which denotes the capacity to effectively cope with life’s challenges and adapt positively to various circumstances. The scale comprises a series of 7 statements, and participants are required to indicate their level of agreement or disagreement with each statement on a Likert scale ranging from 1 (strongly disagree) to 5 (strongly agree).

Typically, the Personal Mastery Scale encompasses statements related to perceptions of personal control, ability to manage stress and difficulties, confidence in problem-solving skills, and a sense of purpose and direction in life. Sample statements may include the following:

1. “I feel in control of my life.”
2. “I am capable of dealing with the challenges and difficulties that life presents to me.”
3. “I have confidence in my ability to solve problems.”
4. “I have a clear sense of purpose and direction in my life.”

The participants rate their agreement with each statement on the Likert scale, and their responses are summed to derive an overall score for personal mastery, reflecting their perceived control over life and their adeptness in coping with challenges. The Personal Mastery Scale has demonstrated validity and reliability, serving as a robust tool for measuring individuals’ perceptions of personal control and adaptability. In this study, the Personal Mastery Scale demonstrated acceptable internal consistency with a Cronbach’s alpha of 0.670, which is considered suitable given the exploratory nature and specific healthcare context of this research. This level of reliability supports its application in assessing personal mastery within the study parameters.

Moreover, this scale has found extensive application in both research and practical settings to explore the pivotal role of mastery in enhancing individual well-being and accomplishing personal and professional aspirations. A multitude of empirical studies have corroborated its efficacy as an assessment instrument for personal mastery, affirming its utility and relevance.

To ensure the rigor of this study, consistent protocols were followed during both data collection and analysis. All questionnaires were administered in a standardized manner, and pilot testing was conducted to validate the survey instruments. We also addressed potential biases by employing random sampling techniques and controlling for confounding variables such as age and years of experience. These measures were critical in enhancing the reliability and validity and credibility of the findings.

## 3. Results

The sample analyzed comprised 27 coordinators (73.0%) and 10 participants in organizational positions (27.0%). From a gender perspective, the group was evenly split between 19 women (51.4%) and 18 men (48.6%). The most common postgraduate education level was a first-level master’s degree for 18 participants (48.6%), followed by a master’s degree for 14 participants (37.8%). In the Italian education system, a Master Level 1 is a postgraduate qualification obtained after completing a bachelor’s degree, whereas a Master Level 2 requires the completion of a master’s degree or an equivalent qualification. This structure may differ from the typical higher education systems in other countries, in which a master’s degree generally refers to a single, unified qualification.

Regarding marital status, the majority of the 23 participants were married (62.2%), while 7 were cohabiting (18.9%) and 4 were divorced (10.8%). Most participants, 31 (83.8%), had children, predominantly two children each for 21 participants (56.8%). Finally, job satisfaction was positive overall, with 16 participants (43.2%) declaring themselves “quite” satisfied and 9 (24.3%) “satisfied” (Table 1).

The participants had a wide range of experience in their current profession, with a minimum of 1 year and a maximum of 42 years, for an average of 19.92 years (SD = 12.678). They had been practicing in their current business unit for a minimum of 1 year and a maximum of 37 years, with a mean of 10.00 years (SD = 10.366). The ages of the participants ranged from 30 to 66 years old, with a mean age of 53.08 years (SD = 7.477). The distance from their home to their workplace varied considerably, from 1 to 40 kilometers, with an average of 13.32 km (SD = 11.785). The time it took them to commute to their workplace was also diverse, ranging from 1 to 60 min, with a mean of 22.19 min (SD = 16.057) (Table 2).

The analysis of personal mastery levels revealed an interesting pattern across the different roles. Coordinators reported a mean personal mastery score of 23.81, indicating relatively high levels of personal agency, self-determination, and ability to shape their work environment. In contrast, participants in organizational positions exhibited a slightly lower mean personal mastery score of 22.60. While the difference in personal mastery between the two groups is not statistically significant, the data suggest that coordinators may possess a stronger sense of personal control and mastery than those in higher-level organizational positions. This finding is counterintuitive, as one might expect those in more senior roles to display more excellent personal mastery skills.

One possible explanation for this result is that the coordinator role’s day-to-day responsibilities and hands-on nature may foster a stronger personal locus of control and self-efficacy. Conversely, the broader strategic focus and greater organizational demands faced by those in organizational positions could lead to a slightly diminished sense of personal mastery, even if the overall levels remain high.

Nonetheless, the data indicate that both coordinators and participants in organizational positions exhibited relatively elevated personal mastery scores, suggesting that individual characteristics related to self-determination and agency may be similar across these distinct leadership roles. Further investigation would be needed to fully understand the nuances underlying these personal mastery dynamics within the healthcare organizational context.

The findings regarding job satisfaction within the examined sample revealed exciting differences between coordinators and participants in organizational positions. The coordinators’ mean job satisfaction score was 3.25, indicating an overall “moderate satisfaction” according to the adopted interpretative ranges. This suggests that, while generally satisfied with their work, the coordinators needed to reach higher satisfaction levels. In stark contrast, the participants in organizational positions reported a mean score of 3.80 on the job satisfaction scale. This value indicates a “high satisfaction” level, demonstrating that this group of professionals exhibited very positive overall satisfaction with their employment.

This marked difference between the two organizational roles could be attributed to several factors. Higher-level leadership positions, such as those held by participants in organizational positions, are often perceived as more prestigious and rewarding, offering greater decision-making autonomy, growth opportunities, and influence over organizational outcomes. Such intrinsic characteristics of senior-level roles translate into generally higher job satisfaction than those observed among coordinators who occupy middle-management positions.

These results underscore the importance of considering the organizational context and the peculiarities of different managerial roles when studying employee job satisfaction. This will help us gain a more in-depth understanding of the factors influencing this construct within healthcare organizations.

The data show differences in job satisfaction and personal mastery between coordinators and those in organizational positions. Coordinators had a lower mean job satisfaction score of 3.25 (SD = 0.716) than those in organizational positions, with a higher mean score of 3.80 (SD = 0.434). This difference was statistically significant (F = 5.086, *p* = 0.030), indicating that those in organizational positions reported higher levels of job satisfaction than coordinators.

In contrast, the coordinators had a higher mean personal mastery score of 23.81 (SD = 4.541) than those in organizational positions, who had a mean of 22.60 (SD = 4.427). However, this difference was not statistically significant (F = 0.529, *p* = 0.472), suggesting that the two groups’ personal mastery levels were similar.

Overall, these results highlight that, while coordinators had lower job satisfaction than those in organizational positions, their levels of personal mastery were comparable. This suggests potential differences in factors influencing job attitudes versus personal characteristics between the two leadership roles (Table 3).

The results show different leadership styles between coordinators and those in organizational positions. Coordinators had higher mean scores in intellectual stimulation (6.15 vs. 5.80), individualized consideration (6.52 vs. 6.30), and management by exception (6.67 vs. 7.20), indicating a more active approach oriented toward employee development. On the other hand, participants in organizational positions reported higher mean scores in idealized influence (6.60 vs. 5.78) and inspirational motivation (6.10 vs. 5.78), suggesting a more charismatic and visionary leadership style. Both groups showed low scores in the “laissez-faire” style, with coordinators having a mean score of 2.44 compared to 3.50 for organizational positions. Overall, these results indicate differences in the way coordinators and managers in organizational positions exercise leadership within the organization (Table 4).

The results of the correlation analysis revealed several significant relationships between the variables considered. Firstly, no significant correlation was found between the mean personal mastery and the other leadership dimensions. However, a positive and significant correlation emerged between mean job satisfaction and management by exception (r = 0.391, *p* < 0.05), suggesting that the leader tends to rely more on an exception-based management approach as job satisfaction increases. The results show that idealized influence was positively and significantly correlated with inspirational motivation (r = 0.650, *p* < 0.01), intellectual stimulation (r = 0.378, *p* < 0.05), individualized consideration (r = 0.334, *p* < 0.05), and management by exception (r = 0.403, *p* < 0.05). This suggests that leaders who exhibit greater idealized influence also tend to score higher on other key dimensions of transformational leadership. Similarly, inspirational motivation was positively correlated with intellectual stimulation (r = 0.497, *p* < 0.01), individualized consideration (r = 0.527, *p* < 0.01), contingent reward (r = 0.416, *p* < 0.05), and management by exception (r = 0.453, *p* < 0.01), indicating a consistent increase across leadership dimensions as inspirational motivation strengthens (Table 5).

The results of the multiple linear regression analysis showed that the overall model explained a significant portion of the variance in job satisfaction within the sample. Among the independent variables considered, two aspects of leadership style demonstrated a statistically significant impact. Specifically, the idealized influence of the leader had a negative and significant effect on job satisfaction (β = −0.609, *p* < 0.001). This suggests that a leadership approach characterized by a high idealized influence is associated with lower levels of employee satisfaction. In contrast, the “Laissez-faire” leadership style, characterized by low leader involvement, showed a positive and significant effect on job satisfaction (β = 0.332, *p* < 0.05). This finding indicates that a more permissive and less directive leadership approach may be associated with greater satisfaction among the nursing coordinators and organizational positions analyzed. The other aspects of leadership style, sense of personal mastery, and average job satisfaction did not demonstrate a statistically significant effect on the dependent variable. Overall, these results suggest that the supervisor’s leadership style can play an important role in determining the levels of job satisfaction among healthcare professionals (Table 6).

## 4. Discussion

The multiple linear regression analysis results showed that the model explained a significant portion of the variance in job satisfaction, which was the dependent variable in this study. Among the independent variables considered, two leadership styles had a statistically significant impact. Specifically, idealized influence demonstrated a negative and significant effect on job satisfaction (β = -0.609, *p* < 0.001), indicating that leaders characterized by a high idealized influence may contribute to lower levels of employee satisfaction. Conversely, the laissez-faire leadership style showed a positive and significant effect on job satisfaction (β = 0.332, *p* < 0.05), suggesting that a more permissive and less directive leadership approach is associated with higher satisfaction levels among the nursing coordinators and organizational positions analyzed. The other leadership dimensions, as well as personal mastery and mean job satisfaction, did not demonstrate statistically significant relationships with job satisfaction. These findings emphasize the significant role that leadership style plays in determining the levels of job satisfaction among healthcare professionals.

This study also revealed notable differences between coordinators and those in organizational positions in terms of job satisfaction. Participants in organizational roles reported higher job satisfaction levels compared to coordinators (F = 5.086, *p* = 0.030). This could be attributed to the perception that higher-level leadership roles provide greater prestige, autonomy, and a sense of accomplishment, which are key factors linked to increased job satisfaction [24,25,26,27]. However, no significant differences were found between the two groups in terms of personal mastery (F = 0.529, *p* = 0.472), suggesting that personal mastery may not be dependent on the leadership role but is likely a reflection of individual characteristics, such as personal agency and the ability to shape one’s work environment, which are known predictors of personal mastery [28,29,30].

In terms of leadership styles, the analysis indicated that coordinators tend to adopt a more transactional approach, characterized by higher scores in intellectual stimulation, individualized consideration, and management by exception, all of which focus on maintaining order and fostering employee development. On the other hand, participants in organizational positions reported higher scores in idealized influence and inspirational motivation, which align more closely with a transformational leadership style focused on vision and charisma [31,32,33]. These differences in leadership styles may reflect the distinct challenges associated with each role. Coordinators, who are often responsible for managing day-to-day operations, may find transactional leadership more effective in maintaining efficiency. In contrast, higher-level leaders, tasked with providing strategic direction and inspiring their teams, may benefit from a more transformational approach [34].

These findings are consistent with the existing literature, which suggests that higher-level leaders are more likely to adopt a transformational leadership style, while lower-level leaders often lean toward transactional leadership [35,36]. This is likely due to the nature of their respective roles, with coordinators focusing on operational efficiency and organizational leaders tasked with setting long-term goals and inspiring staff [37]. This study confirms that the leadership style adopted by healthcare professionals can significantly influence job satisfaction, highlighting the need for tailored leadership development programs that consider the specific roles and responsibilities of nursing leaders.

### Limitations

One fundamental limitation of this study is the relatively small sample size of coordinators and organizational participants examined. A larger and more diverse sample would be needed to enhance the representativeness and generalizability of the findings. Additionally, this research was conducted in a specific organizational context, which restricts the ability to generalize the results to other settings or sectors. Replication of this study in different environments would be necessary to assess the robustness of the results.

Furthermore, the cross-sectional design of this study precludes the establishment of causal relationships between the variables. A longitudinal approach could provide more in-depth insights into the dynamic evolution of job satisfaction and leadership styles over time. Moreover, the exclusive reliance on self-report measures may introduce biases related to self-evaluation and social desirability. Including more objective measures or external assessments could increase the reliability of the findings.

Finally, the interpretations of the observed associations are limited by the inability to determine the direction of causality. Further investigations are needed to understand better the underlying mechanisms that drive the relationships identified in this study. Overall, these limitations indicate the need for further research to validate and expand upon these findings, aiming for a more robust and generalizable understanding of the organizational dynamics explored.

Another limitation of this study is the use of the Personal Mastery Scale (PMS), which, while effective in measuring general aspects of personal mastery, may not be fully specific to workplace dynamics. Future research could benefit from using a tool more directly aligned with workplace-specific measures of personal mastery.

## 5. Conclusions

This study highlights job satisfaction and leadership styles differences between coordinators and organizational participants. Higher-level leaders reported greater job satisfaction, likely due to the prestige and rewards of their roles. Despite this, both groups showed similar levels of personal mastery. Coordinators favored a more active, employee-development approach, scoring higher in intellectual stimulation and individualized consideration, while organizational participants leaned toward charismatic and visionary styles with higher scores in idealized influence and inspirational motivation. This aligns with the literature suggesting that higher-level leaders prefer transformational leadership and lower-level leaders prefer transactional styles [35,38]. A positive correlation was found between job satisfaction and management by exception, indicating that job satisfaction influences the leadership style adopted.

### Implications for Nursing Practice

The insights gained from this study have several practical implications for organizations and personnel management practices, specifically within the research setting. First, the differences in job satisfaction and leadership styles between coordinators and organizational participants highlight the importance of tailoring management and development strategies to employees’ specific needs and characteristics at different levels of the organizational hierarchy. Given the relatively small sample size, further research is needed to confirm the generalizability of these findings across different contexts.

Secondly, the finding that job satisfaction can shape leadership styles underscores the significance of fostering a positive work environment and promoting employee well-being. By enhancing job satisfaction, organizations may be able to encourage the adoption of more effective and employee-centric leadership approaches, which can ultimately contribute to improved organizational performance and employee engagement.

Finally, this study’s results emphasize the need for ongoing training and development programs addressing the unique challenges and responsibilities of different leadership roles. By equipping managers with the necessary skills and knowledge to lead their teams effectively, organizations can optimize their personnel management practices and foster a more productive and harmonious work environment.

## Figures and Tables

**Table 1:** Demographic characteristics of the analyzed sample.

**Role**		
	*n*	%
Coordinator	27	73.0
Organizational position	10	27.0
**Gender**		
Female	19	51.4
Male	18	48.6
**Post-Basic Education**		
Master’s degree	14	37.8
Master 1° level	18	48.6
Master 2° level	3	8.1
Other degree	2	5.4
**Marital Status**		
Co-habitation	7	18.9
Divorced	4	10.8
Single	1	2.7
Married	23	62.2
Widower	2	5.4
**Children**		
No	6	16.2
Yes	31	83.8
**How many children?**		
0	6	16.2
1	5	13.5
2	21	56.8
3	2	5.4
4	1	2.7
ND	2	5.4
**How satisfied you are with your work?**		
By no means	1	2.7%
Little	5	13.5%
Enough	16	43.2%
Satisfied	9	24.3%
A lot	6	16.2%

**Table 2:** Descriptive statistics of professional and commuting characteristics.

	Min	Max	Mean	SD
How long have you been in your current profession?	1	42	19.92	12.678
How long have you been practicing in your current business unit?	1	37	10.00	10.366
Age	30	66	53.08	7.477
How far is your work from home? (in km)	1	40	13.32	11.785
How long does it take you to get to your workplace? (in minutes)	1	60	22.19	16.057

**Table 3:** Job satisfaction and personal mastery by role.

		Mean Job Satisfaction	Mean Personal Mastery
Nurse coordinator	Mean	3.25	23.81
	*n*	27	27
	SD	0.716	4.541
Organizational position	Mean	3.80	22.60
	*n*	10	10
	SD	0.434	4.427
Total	Mean	3.40	23.49
	*n*	37	37
	SD	0.691	4.482
F		5.086	0.529
*p*-value		0.030	0.472

**Table 4:** Leadership styles by role.

Leadership Style Dimensions								
		Idealized Influence	Inspirational Motivation	Intellectual Stimulation	Individualized Consideration	Contingent Reward	Management by Exception	Laissez-Faire
Nurse coordinator	Mean	5.78	5.78	6.15	6.52	5.74	6.67	2.44
	*n*	27	27	27	27	27	27	27
	SD	1.761	1.450	1.634	1.762	1.873	1.819	1.695
Organizational position	Mean	6.60	6.10	5.80	6.30	6.10	7.20	3.50
	*n*	10	10	10	10	10	10	10
	SD	1.430	1.969	1.549	2.669	1.912	1.874	2.461
Total	Mean	6.00	5.86	6.05	6.46	5.84	6.81	2.73
	*n*	37	37	37	37	37	37	37
	SD	1.700	1.584	1.598	2.008	1.864	1.823	1.953
F		1.743	0.296	0.340	0.084	0.266	0.618	2.203
*p*-value		0.195	0.590	0.563	0.773	0.609	0.437	0.147

**Table 5:** Correlations between personal mastery, job satisfaction, and leadership dimensions (*n* = 37).

Leadership Style Dimensions										
		Mean Personal Mastery	Mean Job Satisfaction	Idealized Influence	Inspirational Motivation	Intellectual Stimulation	Individualized Consideration	Contingent Reward	Management by Exception	Laissez-Faire
Median personal mastery	r	1								
	Sign									
Median job satisfaction	r	0.196	1							
	Sign	0.246								
Idealized influence	r	0.055	0.277	1						
	Sign	0.748	0.097							
Inspirational motivation	r	0.119	0.307	0.650 **	1					
	Sign	0.483	0.064	0.000						
Intellectual stimulation	r	0.035	0.097	0.378 *	0.497 **	1				
	Sign	0.837	0.567	0.021	0.002					
Individualized consideration	r	0.243	0.256	0.334 *	0.527 **	0.719 **	1			
	Sign	0.147	0.126	0.044	0.001	0.000				
Contingent reward	r	0.106	0.090	0.254	0.416 *	0.619 **	0.629 **	1		
	Sign	0.532	0.595	0.129	0.010	0.000	0.000			
Management by exception	r	−0.009	0.391 *	0.403 *	0.453 **	0.576 **	0.586 **	0.522 **	1	
	Sign	0.959	0.017	0.013	0.005	0.000	0.000	0.001		
Style of leadership “Laissez-faire”	r	−0.242	0.152	0.167	0.338 *	0.112	0.188	0.255	0.407 *	1
	Sign	0.150	0.368	0.322	0.041	0.511	0.264	0.128	0.013	

*. The correlation is significant at the 0.05 (two-tailed) level. **. The correlation is significant at the 0.01 (two-tailed) level.

**Table 6:** Predictors of job satisfaction among nursing coordinators and organizational positions.

			Standardized Coefficients	t	Sign	95.0% Confidence Interval for B		Correlations			Collinearity Statistics	
			Beta			Lower Limit	Upper Limit	Zero Order	Partial	Part	Tolerance	VIF
(Constant)	6.883	1.709		4.029	0.000	3.378	10.389					
Mean personal mastery	0.016	0.054	0.043	0.299	0.767	−0.096	0.128	−0.111	0.057	0.037	0.768	1.303
Mean job satisfaction	−0.544	0.364	−0.220	−1.493	0.147	−1.290	0.203	−0.319	−0.276	−0.187	0.723	1.383
Idealized influence	−0.611	0.172	−0.609	−3.564	0.001	−0.963	−0.259	−0.651	−0.566	−0.447	0.539	1.857
Inspirational motivation	−0.215	0.211	−0.200	−1.023	0.315	−0.647	0.217	−0.432	−0.193	−0.128	0.412	2.430
Intellectual stimulation	0.113	0.227	0.105	0.497	0.623	−0.352	0.578	−0.134	0.095	0.062	0.349	2.864
Individualized consideration	0.138	0.182	0.163	0.761	0.453	−0.235	0.512	−0.098	0.145	0.095	0.343	2.915
Contingent reward	−0.141	0.162	−0.154	−0.870	0.392	−0.472	0.191	−0.109	−0.165	−0.109	0.504	1.983
Management by exception	0.083	0.180	0.088	0.457	0.651	−0.288	0.453	−0.123	0.088	0.057	0.423	2.364
Leadership style “Laissez-faire”	0.290	0.135	0.332	2.148	0.041	0.013	0.567	0.158	0.382	0.269	0.658	1.521

Note: Dependent variable: How satisfied are you with your job? Note: The first column shows the unstandardized coefficients (B) for each variable, and the second column provides the corresponding standard errors. The R^2^ value represents the proportion of variance explained by the model.

## Data Availability

The data presented in this study are available upon request from the corresponding author.

## Public Involvement Statement

There was no public involvement in any aspect of this research.

## Guidelines and Standards Statement

This manuscript was prepared in accordance with the STROBE (Strengthening the Reporting of Observational Studies in Epidemiology, Von elm et al., 2014) guidelines for reporting observational studies.
